# The impact of a simple positioning aid device on the diagnostic performance of thyroid cancer in CT scans: a randomized controlled trial

**DOI:** 10.1186/s40644-025-00878-w

**Published:** 2025-05-08

**Authors:** Wei-Hua Lin, Hui-Juan Huang, Wen-Cong Yang, Qing-Wen Huang, Rui-Gang Huang, Fu-Rong Luo, Dong-Yi Chen, Zheng-Han Yang, Hai-Tao Li, Hui-Huang Zeng, Hui-Jun Xiao

**Affiliations:** 1 Department of Radiology, Zhangzhou Affiliated Hospital of Fujian Medical University, Zhangzhou, China; 2 Department of Radiology, Beijing Friendship Hospital, Capital Medical University, Beijing, China

**Keywords:** Positioning aid device, Thyroid CT scan, Thyroid cancer, Diagnostic performance, Image quality

## Abstract

**Objective:**

To evaluate the effectiveness of a simple positioning aid device in neck CT scans for the diagnosis of thyroid cancer, with a focus on its influence on image quality and diagnostic accuracy.

**Methods:**

A randomized controlled trial was conducted involving 180 patients with suspected thyroid cancer. Participants were randomly assigned to two groups: the device-assisted positioning group (Group A) and the traditional positioning group (Group B). A total of 147 patients who underwent enhanced neck CT scans and subsequent surgical pathological biopsies were included in the final analysis. Image quality and thyroid disease diagnoses were independently assessed by two experienced radiologists, with a unified consensus for the final conclusions. Objective imaging parameters and subjective ratings were used to evaluate image quality. Pathological findings served as the gold standard to compare the diagnostic accuracy of the two groups for thyroid malignancy, capsular invasion, and lymph node metastasis. Additionally, radiation doses in both groups were compared.

**Results:**

A total of 147 patients were included in the analysis, with 72 patients in Group A and 75 in Group B. The baseline characteristics of the two groups were similar (*P* > 0.05). Group A demonstrated significantly superior image quality compared to Group B, with shorter length of artifacts (LA), lower proportion of affected thyroid (PA), and lower artifact index (AI). Subjective assessments also favored Group A, showing better ratings for regional artifacts and overall image quality. In terms of diagnostic accuracy, Group A outperformed Group B in detecting thyroid cancer (AUC: 0.852 vs. 0.676, *P* = 0.021). For the right thyroid lobe, Group A had significantly better diagnostic performance (AUC: 0.897 vs. 0.746, *P* = 0.016). Group A also showed superior performance in diagnosing capsular invasion (AUC: 0.861 vs. 0.721, *P* = 0.037), with similar results observed for both the left and right thyroid lobes. There was no significant difference between the groups in diagnosing lymph node metastasis. Furthermore, thyroid region radiation doses (CTDIvol and SSDE) were significantly lower in Group A compared to Group B.

**Conclusion:**

The use of a positioning aid device significantly improves CT image quality, enhancing diagnostic accuracy for malignant thyroid lesions and capsular invasion, while also reducing radiation exposure.

**Supplementary Information:**

The online version contains supplementary material available at 10.1186/s40644-025-00878-w.

## Introduction

In recent years, the prevalence of thyroid nodules has risen to approximately 65%, with 5–15% of these nodules being malignant [1, 2, 3]. Accurately distinguishing between benign and malignant nodules is critical to avoid unnecessary treatment. For thyroid cancer, identifying capsular invasion and lymph node metastasis is vital for treatment planning and prognosis evaluation. While biopsy and surgical pathology remain the gold standards for diagnosis, they are invasive, carry inherent risks, and have limitations in assessing tumor heterogeneity [4] Consequently, non-invasive imaging techniques are of significant interest for improving diagnostic accuracy.

Ultrasound is the primary imaging modality for diagnosing thyroid cancer and assessing cervical lymph nodes preoperatively [5] However, its ability to visualize deep structures can be hindered by interference from bone or gas, leading to limitations in detecting lymph node metastases, with only 38-59% of cases identified initially [6] For patients with suspected local progression or lymph node metastasis, contrast-enhanced CT (CECT) is recommended due to its superior anatomical resolution and spatial visualization [7, 8, 9], playing a key role in preoperative tumor staging [10, 11, 12]. However, traditional neck CT scans can be compromised by forward displacement of the shoulders, which may partially obscure the thyroid region, and the presence of dental fillings, which can induce beam hardening and photon starvation artifacts. These artifacts may distort or obscure critical information, particularly when evaluating the lower thyroid lobes and adjacent deep cervical lymph nodes, primarily involving levels VI, IV, and V, thereby diminishing diagnostic accuracy [13, 14, 15].

While much of the research has focused on scanning techniques and reconstruction methods to minimize artifacts, the role of patient positioning has been largely overlooked. Preliminary clinical practice at our institution has shown that elevating the neck and lowering the shoulders during neck CT scans can reduce strip artifacts in the thyroid region. However, this approach has been sparsely reported in the literature. To stabilize this positioning, we developed a simple positioning aid device aimed at improving image quality and enhancing diagnostic capability in thyroid cancer detection through neck CT scans.

## Methods

### Study design and participants

This prospective randomized controlled trial was registered with the Chinese Clinical Trial Registry (https://www.chictr.org.cn/, registration number: ChiCTR2400079494) and approved by the Institutional Review Board (2023KYZ187). Informed consent was obtained from all participants. The study enrolled 180 patients with suspected thyroid cancer, based on ultrasound findings, who underwent contrast-enhanced neck CT scans between May and August 2024 at our institution.

**Fig. 1:**
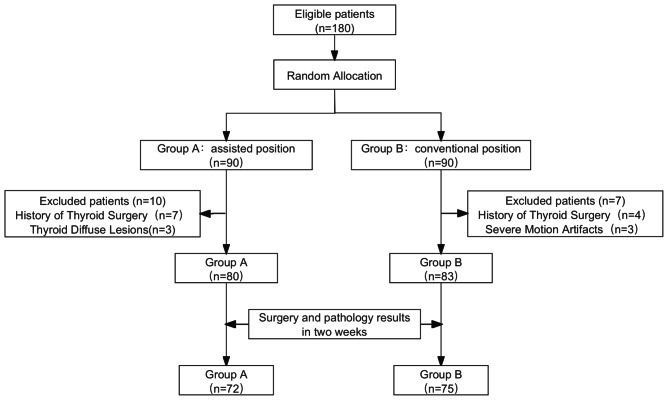
Schematic illustration of patient recruitment and study design

The inclusion criteria were: (1) age ≥ 18 years, capable of following instructions; (2) normal thyroid function; (3) no significant liver or kidney dysfunction; (4) non-pregnant. Exclusion criteria included a history of thyroid surgery, severe motion artifacts, or other conditions that could interfere with the study. After applying the exclusion criteria, 163 patients underwent CT scanning. A total of 147 patients (the device-assisted positioning group (Group A: 72) and the traditional positioning group (Group B: 75)) proceeded with surgery and biopsy within two weeks post-CT scan, and the corresponding pathological results were obtained. These 147 patients were included in the final diagnostic efficacy analysis (Fig. 1).

Two experienced radiologists independently assessed the image quality and radiological diagnosis. In the case of discrepancies between their assessments, the radiologists discussed the results to reach a consensus. Both radiologists were blinded to the study objectives as well as to the patients’ surgical and pathological outcomes.

### Patient positioning design

**Fig. 2:**
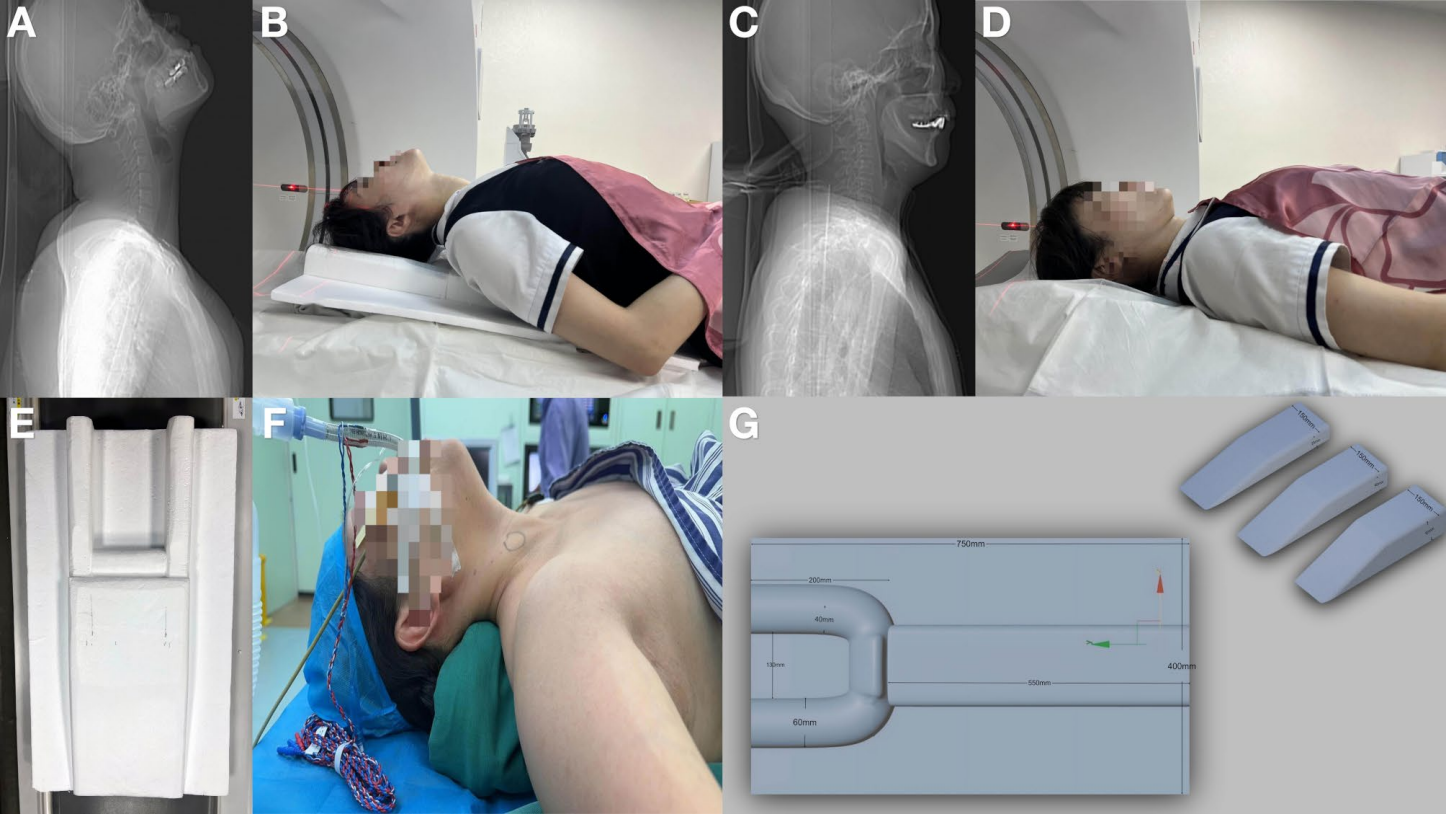
Illustration of the auxiliary device-assisted position, traditional position, surgical position, and auxiliary devices. **(A**,** B)** Representative patient positioning photographs (A) and the scout image **(B)** in Group A with assisted positioning. **(C**,** D)** Representative patient positioning photographs **(C)** and the scout image **(D)** in Group B with traditional positioning. In Group A, the shoulders are lowered, and the neck is elevated, resulting in less overlap between the neck and shoulders in the sagittal view; in the traditional position, the shoulders are moved forward, causing the neck to be obscured by the shoulders in the sagittal view. **(E)** Photograph of the auxiliary device. **(F)** Photograph of the surgical position. **(G)** Structural diagram of the auxiliary device

As depicted in Fig. 2, patients in Group A used a specially designed “Neck CT Positioning Pad” to optimize and stabilize their position during the scan. The pad, made of high-strength foam, was designed to fill the concave areas of the examination table, creating a head-up, feet-down slope that elevated the back. Additionally, the pad featured a U-shaped pillow to stabilize the head. The dimensions of the device components are shown in the figure.

Patients in Group A were positioned supine, with their heads tilted back, and entered the scanner head-first with their arms naturally by their sides. This positioning allowed the shoulders to shift backward into an arch, reducing the horizontal width, and the chin was elevated to move the thyroid gland away from the chest. In contrast, Group B patients were positioned in the traditional supine manner, entering the scanner head-first with arms at their sides, without the use of any supplementary positioning aids.

### CT examination procedure

All CT scans were performed using a 64-slice CT scanner (Revolution Maxima, GE Healthcare), with consistent scanning parameters and contrast injection protocols for both groups. The scanning procedure included three phases: non-contrast scan, arterial phase (25 s post-contrast injection) [16], and venous phase (55 s post-contrast injection). The scanning range extended from the inferior edge of the tracheal bifurcation to the bony structure of the external ear canal. Scanning parameters were: spiral scan, detector width of 64 × 0.625 mm, pitch ratio of 0.516:1, tube voltage of 120 kV, automatic tube current modulation (50 mA to 400 mA), noise index of 8. Images were reconstructed using the 40% ASiR-V algorithm, with a slice thickness and interval of 1.25 mm. The contrast agent (Iodixanol, 400mgI/ml, Patheon Italia S.P.A.) was injected intravenously via a high-pressure injector (XD200x, Ulrich Medical, Germany) into the antecubital vein over 20 s. The contrast agent dose was calculated using the following formula:

Contrast agent volume = [Weight (kg) × 340 (mgI/kg)] / 400 (mgI/ml) [17]. Subsequently, saline was injected at the same rate over 10 s.

### Image quality evaluation

#### Objective image quality

Venous-phase images were analyzed using the AW 4.7 workstation. Regions of interest (ROI) were placed to avoid the lesion area and covered two-thirds of the tissue. Each structure was measured three times, and the average was taken as the final result. The study measured the maximum length of thyroid artifacts (LA), the SD values of the most affected and least affected regions of the thyroid, and calculated other objective image quality parameters, including PA, AI, SNR, and CNR, as follows:

For LA measurement, on the venous-phase coronal reconstructed images, the layer showing thyroid artifacts was selected, and the maximum craniocaudal diameter of the continuous artifact region on that layer was measured. Each patient was measured three times, and the average value was used. To further standardize the evaluation, we calculated the ratio between artifact length and the actual length of the thyroid lobe: the maximum craniocaudal length of the artifact region was divided by the maximum craniocaudal length of the ipsilateral thyroid lobe on the same coronal slice. (Lthyroid)

The most affected region (SDmax) was defined as the ROI within the thyroid tissue that showed significant artifact interference and inhomogeneous texture, while the least affected region (SDmin) was selected from an area with uniform density and minimal artifact influence. Each ROI covered approximately two-thirds of the thyroid area on the same slice, avoided any lesions, and was measured three times to obtain the mean value.

1. Proportion of the length of thyroid affected by artifacts (PA) = LA / Lthyroid * 100%.
2. Artifact Index (AI) = (SDmax² - SDmin²)¹/².
3. Signal-to-noise ratio (SNR) = HU / SD.
4. Contrast-to-noise ratio (CNR) = (HUthyroid - HUmuscle) / SDmuscle.

#### Subjective image quality

1. Artifact scoring (4-point scale): Artifacts in the upper, middle, and lower lobes of the thyroid, and the isthmus were scored as follows: 1 = severe artifacts, 2 = moderate artifacts, 3 = mild artifacts, 4 = no artifacts.
2. Overall image quality scoring (5-point scale): The overall image quality of the neck and thyroid was rated as follows: 1 = poor image quality, not suitable for diagnosis; 2 = suboptimal quality, limited detail recognition and interpretation; 3 = acceptable quality, suitable for basic diagnosis; 4 = good quality, suitable for detailed diagnosis; 5 = excellent quality, providing clear anatomical details suitable for high-quality diagnosis. Scores ≥ 2 were considered acceptable for diagnosis (all images in this study had a score ≥ 2) [18].

### Diagnostic efficacy evaluation

Diagnosis was primarily based on the American Thyroid Association (ATA) Guidelines. Given the absence of a standardized classification system specifically for CT-based thyroid imaging, this study referred to the sonographic morphological criteria described in the American College of Radiology Thyroid Imaging Reporting and Data System (ACR TI-RADS) and related Radiopaedia guidelines [19, 20, 21], including features such as margin, attenuation, calcification, and enhancement pattern, to assist in the evaluation of imaging characteristics potentially associated with malignancy. However, due to the inherent differences in image presentation between ultrasound and CT, certain features such as microcalcifications may not be directly translatable and were interpreted with caution.

Malignancy, capsular invasion, and lymph node metastasis were scored using a 4-point scale: 1 = “definitely negative,” 2 = “probably negative,” 3 = “probably positive,” and 4 = “definitely positive.” Scores ≥ 3 were considered positive results. Imaging findings were compared with the pathological gold standard, and the area under the receiver operating characteristic curve (AUC) was calculated. A higher AUC indicates superior diagnostic efficacy. Additional diagnostic performance metrics, such as sensitivity, specificity, accuracy, positive predictive value, and negative predictive value, were also assessed.

### Radiation dose assessment

Following the non-contrast scan, axial images were used to manually define the scan range, and the estimated CTDIvol (Computed Tomography Dose Index Volume) for the venous-phase thyroid and the entire neck was automatically recorded. The venous-phase CTDIvol was directly obtained from the dose report. Furthermore, based on the American Association of Physicists in Medicine (AAPM) report [22], the specific volume dose estimate was calculated using the following formula:

SSDE = ƒed × CTDIvol,

where ƒed (conversion factor) = 3.704369 × e^(-0.03671937×ED)^,

and ED (effective diameter) = (RL * AP)¹/².

### Statistical analysis

Data analysis was performed using SPSS version 23.0 (IBM SPSS Inc., Armonk, NY). Continuous variables with a normal distribution were expressed as mean ± standard deviation (SD) and compared using the independent samples t-test. Non-normally distributed data were presented as median [Q1, Q3] and analyzed using the Mann-Whitney U test. Categorical variables were expressed as frequencies (percentages) and analyzed using the chi-square test. Receiver operating characteristic (ROC) analysis was used to compute the area under the curve (AUC), and the Delong test was applied to compare AUCs. The chi-square test was used for comparisons of other diagnostic parameters. A two-tailed test was used, and statistical significance was set at α < 0.05.

## Results

### Patient characteristics

A total of 147 patients were included in this study, with a mean age of 51.0 [39.5, 58.0] years, and 78.9% were female. Of these, 72 patients were in Group A, and 75 patients were in Group B. As shown in Table 1, there were no statistically significant differences between the two groups regarding gender, age, BMI, or contrast agent usage (all *P* > 0.05).

**Table 1:** Patient characteristics

Characteristics	Overall (*n* = 147)	Group A (*n* = 72)	Group B (*n* = 75)	*P* value
Age (years)	51.0 [39.5, 58.0]	49.5 [35.5, 55.0]	54.0 [42.0, 59.5]	0.091
Female	116 (78.9)	61 (84.7)	55 (73.3)	0.136
Height (cm)	159.0 [156.0, 164.0]	159.0 [156.0, 163.0]	160.0 [156.0, 165.0]	0.876
Weight (kg)	60.0 [54.5, 68.5]	60.0 [53.0, 66.0]	62.0 [55.0, 70.0]	0.304
Body mass index, BMI (kg/m^2^)	23.5 [21.2, 26.3]	23.3 [20.9, 25.9]	23.9 [21.3, 26.6]	0.389
Contrast dose (mL)	52.0 [47.5, 59.0]	52.0 [46.0, 56.0]	54.0 [48.0, 60.0]	0.246
Injection rate (mL/s)	2.6 [2.4, 2.9]	2.6 [2.3, 2.8]	2.7 [2.4, 3.0]	0.213

Continuous variables are presented as median [upper quartile, lower quartile] and categorical variables are expressed as *n* (%)

### Image quality evaluation

The objective imaging quality parameters and evaluation indices are presented in Table 2. The LA, PA, and AI of the left and right thyroid lobes in Group A were significantly lower than those in Group B (all *P* < 0.001). Except for the CNR of the least affected region by artifacts, which showed no difference between Groups A and B, the SNR in both the most and least affected regions of the left and right thyroid lobes was significantly higher in Group A than in Group B (all *P* < 0.05). The CNR in the most affected region by artifacts was also significantly better in Group A compared to Group B (all *P* < 0.05).

**Table 2:** The objective image quality between two groups

Parameters	Group A (*n* = 72)	Group B (*n* = 75)	*P* value
**Left thyroid**			
L_A_ (mm)	0.0 [0.0, 6.2]	18.9 [12.0, 32.5]	< 0.001
P_A_ (%)	0.0 [0.0, 11.8]	42.2 [24.5, 63.2]	< 0.001
AI	5.7 [3.9, 7.6]	14.4 [11.0, 21.0]	< 0.001
Heaviest artifact affected area			
SD	9.0 [8.0, 10.0]	17.0 [13.0, 23.0]	< 0.001
SNR	16.7 [13.7, 19.0]	7.1 [5.6, 9.3]	< 0.001
CNR	11.0 [8.2, 13.5]	7.3 [4.3, 10.2]	< 0.001
Least artifact affected area			
SD	6.0 [6.0, 7.0]	7.0 [6.0, 8.0]	0.006
SNR	23.1 [21.2, 26.6]	22.0 [18.7, 24.8]	0.024
CNR	14.2 [11.5, 17.0]	13.4 [10.5, 15.3]	0.059
**Right thyroid**			
L_A_ (mm)	0.0 [0.0, 0.6]	18.9 [9.4, 32.5]	< 0.001
P_A_ (%)	0.0 [0.0, 1.3]	39.1 [19.3, 61.0]	< 0.001
AI	5.7 [3.9, 8.0]	14.8 [10.3, 21.1]	< 0.001
Heaviest artifact affected area			
SD	8.5 [8.0, 10.0]	17.0 [13.0, 23.0]	< 0.001
SNR	16.5 [13.3, 19.6]	6.7 [5.3, 9.1]	< 0.001
CNR	11.9 [8.8, 14.5]	6.5 [3.7, 10.6]	< 0.001
Least artifact affected area			
SD	6.0 [6.0, 7.0]	7.0 [6.0, 8.0]	< 0.001
SNR	23.3 [21.4, 25.5]	21.3 [18.5, 23.3]	< 0.001
CNR	14.4 [11.8, 16.2]	13.2 [10.7, 16.1]	0.065

**Fig. 3:**
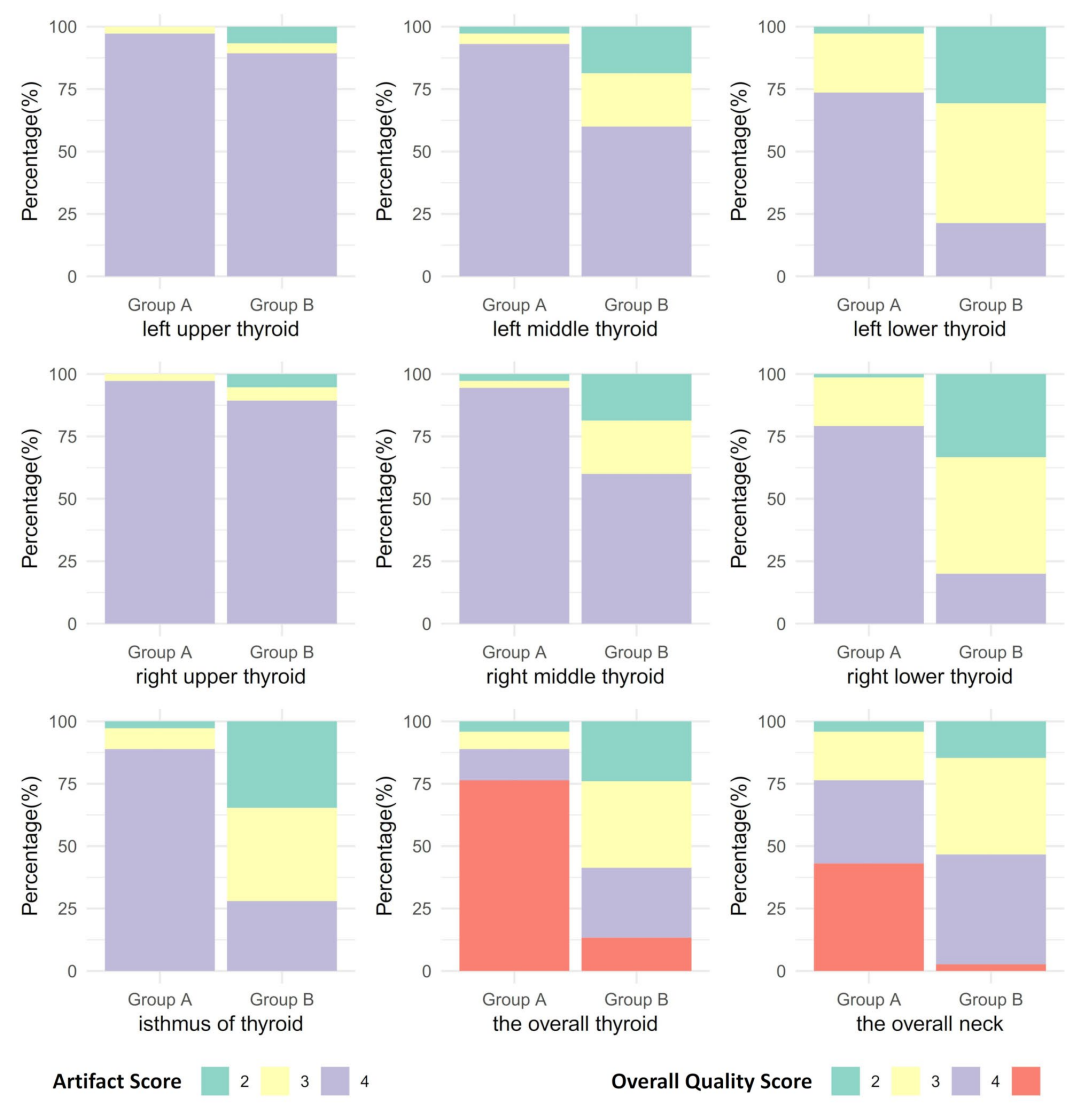
The subjective image quality scores in both groups

The subjective image quality scores are shown in Fig. 3. Except for the scores of the upper poles of the left and right thyroid lobes, where there was no significant difference between Groups A and B (*P* = 0.05, with over 90% of scores being 4), the scores for other regions (left and right thyroid middle and lower poles, and isthmus) were significantly higher in Group A than in Group B (all *P* < 0.001). The median overall image quality scores for the thyroid and neck in Group A were 5 and 4, respectively, which were significantly higher than those in Group B (both 3, *P* < 0.001).

### Diagnostic efficacy evaluation

A total of 147 patients ultimately underwent pathological examination. The pathological diagnosis of thyroid cancer positivity rate (Group A: 70.8%, Group B: 64.0%, *P* = 0.479), capsular invasion positivity rate (Group A: 50.0%, Group B: 48.0%, *P* = 0.938), and lymph node metastasis positivity rate (Group A: 30.6%, Group B: 26.7%, *P* = 0.735) showed no statistically significant differences between Groups A and B.

**Fig. 4:**
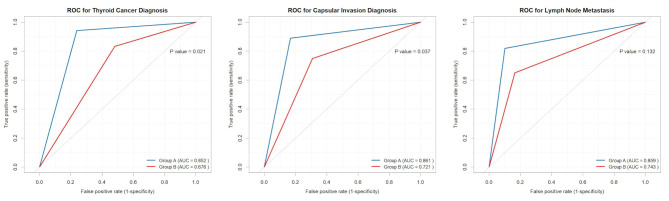
The diagnostic efficacy for thyroid cancer, capsular invasion and lymph node metastasis between the two groups

In diagnosing benign and malignant thyroid lesions and capsular invasion (Fig. 4), Group A outperformed Group B. Specifically, the AUC for diagnosing thyroid cancer was 0.852 in Group A and 0.676 in Group B (*P* = 0.021), while the AUC for diagnosing capsular invasion was 0.861 in Group A and 0.721 in Group B (*P* = 0.037). Additionally, the diagnostic accuracy for thyroid cancer in Group A (88.9%) was significantly higher than that in Group B (72.0%) (*P* = 0.018). No significant difference was found in the diagnostic efficacy for lymph node metastasis between the two groups (Fig. 4). Specific diagnostic efficacy-related indicators are shown in Table 3.

**Table 3:** The diagnostic performances in thyroid cancer between two groups

		Group A (*n* = 72)	Group B (*n* = 75)	*P* value
**Malignancy**	Pathological diagnosis positive, *n*	51	48	0.479
	Image diagnosis positive, *n*	53	53	0.831
	AUC	0.852	0.676	0.021
	Sensitivity, %	94.1	83.3	0.166
	Specificity, %	76.2	51.9	0.154
	Accuracy, %	88.9	72.0	0.018
	PPV, %	90.6	75.5	0.070
	NPV, %	84.2	63.6	0.259
**Capsular invasion**	Pathological diagnosis positive, *n*	36	36	0.938
	Image diagnosis positive, *n*	38	39	1.000
	AUC	0.861	0.721	0.037
	Sensitivity, %	88.9	75.0	0.220
	Specificity, %	83.3	69.2	0.247
	Accuracy, %	86.1	72.0	0.058
	PPV, %	84.2	69.2	0.199
	NPV, %	88.2	75.0	0.265
**Lymph node metastasis**	Pathological diagnosis positive, *n*	22	20	0.735
	Image diagnosis positive, *n*	23	22	0.869
	AUC	0.859	0.743	0.132
	Sensitivity, %	81.8	65.0	0.375
	Specificity, %	90.0	83.6	0.503
	Accuracy, %	87.5	78.7	0.228
	PPV, %	78.3	59.1	0.286
	NPV, %	91.8	86.8	0.616

AUC, area under the receiver operating characteristic curve; NPV, negative predictive value; PPV, positive predictive value

The study further analyzed the diagnostic efficacy for thyroid cancer and capsular invasion in different parts of the thyroid (Appendix 1, 2). The results indicated that for diagnosing malignant lesions in the right thyroid lobe, Group A (AUC: 0.897) had significantly higher diagnostic efficacy than Group B (AUC: 0.746), with statistical significance (*P* = 0.016, Appendix 1). In the diagnosis of capsular invasion (Appendix 2), Group A outperformed Group B in both the left thyroid lobe (Group A AUC: 0.912 vs. Group B AUC: 0.750, *P* = 0.043) and the right thyroid lobe (Group A AUC: 0.891 vs. Group B AUC: 0.706, *P* = 0.034), with statistically significant differences.

### Radiation dose evaluation

The median CTDIvol for the thyroid region was 11.2 [8.7, 14.3] in Group A and 12.5 [10.2, 19.3] in Group B (*P* = 0.011). The median SSDE was 13.3 [10.5, 16.5] in Group A and 14.9 [11.7, 21.3] in Group B (*P* = 0.032). Both differences were statistically significant.

## Discussion

In the evaluation of thyroid pathology using cervical CT, device-assisted positioning has been shown to reduce thyroid radiation exposure, minimize artifacts, improve image quality, and enhance radiologists’ diagnostic performance for thyroid cancer malignancy and capsular invasion, with pathological results serving as the reference standard.

Previous studies on thyroid CT artifacts have largely concentrated on image reconstruction and scanning technologies. For example, the use of higher-energy virtual monoenergetic images, hybrid iterative reconstruction, and adaptive filtering techniques in spectral imaging has been reported to reduce streak artifacts in the neck and shoulder regions [23, 24]. Streak artifacts in the thyroid region are primarily caused by beam hardening and photon starvation due to the overlap of thyroid tissue and shoulder bone in the axial plane. In the upper neck, artifacts are mostly attributed to metal objects, such as dental fillings, and other adjacent tissues [25].

In this study, the use of an auxiliary device to optimize positioning significantly reduced artifacts in the thyroid and upper neck regions. The results indicate that image artifacts were significantly reduced in the device-assisted positioning group. The reduction in artifacts and the subsequent improvement in image quality were especially pronounced in the middle and lower thyroid lobes and the isthmus, as these areas are more prone to obstruction by the shoulder. The upper thyroid pole, however, is less affected by shoulder obstruction and is mainly influenced by artifacts from dental fillings, so the device-assisted positioning had less impact on this area. Some studies have used arm traction devices to lower the acromion by an average of 2.1 cm to improve image quality in the lower neck; however, this approach is limited by the patient’s physical endurance and can lead to motion artifacts due to prolonged multi-phase scanning [26].

Due to the reduction in streak artifacts, the overall image quality of the neck and thyroid in the device-assisted positioning group was superior to that in the traditional positioning group. This improvement likely contributed to more accurate diagnoses. While most studies focus on image quality, this study not only explored image quality but also assessed diagnostic performance with histopathology as the reference. Both primary and senior radiologists demonstrated superior performance in diagnosing thyroid malignancies and capsular invasion when using device-assisted positioning. Optimizing positioning to enhance diagnostic capabilities for malignant lesions is essential for treatment decisions, surgical planning, and prognosis assessment in thyroid cancer. However, no statistically significant difference was observed between the two groups in the diagnosis of lymph node metastasis. Although streak artifacts were predominantly located in the lower cervical region in the conventional positioning group—potentially affecting image interpretation in this area—and cases of lower cervical lymph node metastasis were present in our cohort, the limited number of such cases precluded a robust evaluation of imaging performance or diagnostic accuracy in this specific region. Further studies with larger sample sizes are needed to validate these potential effects.

It is also important to note that certain regions of the thyroid, such as the isthmus, have a limited number of nodules, which could affect the reliability of diagnostic performance evaluations. This may explain why, in some diagnostic parameters, the traditional positioning group outperformed the device-assisted positioning group.

The device-assisted positioning also reduced thyroid radiation exposure through automatic exposure control technology, decreasing the specific volume dose estimate (SSDE) by 15.10%. In traditional positioning, higher mA and radiation doses are often used to compensate for predictable photon starvation artifacts to ensure adequate image quality. However, device-assisted positioning allows for acceptable image noise at lower radiation doses. Although thyroid radiation doses were not directly obtained from the dose reports in this study, manual estimates of cervical doses showed no statistical difference compared to the dose report values, which is why manual estimates were used to assess thyroid dose.

Additionally, the device-assisted positioning is similar to the thyroid surgical position (as shown in Fig. 2F), which may help surgeons more accurately locate and identify lesions before surgery. Furthermore, clearer preoperative images assist in identifying and avoiding damage to critical structures, such as the parathyroid glands, during surgery, thereby reducing the risk of intraoperative injury. In practice, we did not recommend patients avoid swallowing, as such advice could increase the risk of motion artifacts from swallowing. Interestingly, during the inclusion and exclusion process, no patients in Group A were excluded due to motion artifacts, while three patients in Group B were excluded due to severe swallowing artifacts. This suggests that patients in Group A, due to their extended neck position, found it more difficult to swallow, which may have contributed to a reduction in swallowing-induced motion artifacts. This may be related to factors such as cervical muscle tension, changes in neural pathways, anatomical variations in the esophagus, and delayed swallowing reflexes [27, 28, 29, 30].

### Limitations

This study has several limitations. First, the single-center design and relatively small sample size limit the generalizability of the findings and restrict the ability to conduct subgroup analyses. Second, thyroid radiation dose was estimated based on pre-scan imaging, and future studies should use specialized equipment to directly assess thyroid radiation exposure. Furthermore, the auxiliary positioning device should be further refined through large-scale studies to accommodate the individualized needs of patients with varying body types and ages, while improving comfort, ease of use, and compatibility with CT scanners from multiple manufacturers. It should be noted that in clinical practice, patients with restricted cervical mobility or shoulder dysfunction may still experience difficulty adapting to the device, suggesting that future designs should better address compatibility with special functional limitations.

## Conclusion

The use of device-assisted positioning during cervical CT scans can reduce thyroid radiation exposure, decrease artifacts, enhance image quality, and may improve the accuracy of thyroid cancer diagnosis.

## Electronic supplementary material

Below is the link to the electronic supplementary material.

Supplementary Material 1

Supplementary Material 2

## Data Availability

No datasets were generated or analysed during the current study.
