# Supplementary material for: The impact of a simple positioning aid device on the diagnostic performance of thyroid cancer in CT scans: a randomized controlled trial
Source: Cancer Imaging. 2025 May 8;25:60. doi: 10.1186/s40644-025-00878-w (PMC12063306; doi:10.1186/s40644-025-00878-w)
Supplement: Supplementary file 1 — Supplementary Material 1 [file 40644_2025_878_MOESM1_ESM.doc]

**Supplemental Table 1. The diagnostic performances in capsular invasion of thyroid cancer**

|  | Group A (n=72) | Group B (n=75) | P value |
| --- | --- | --- | --- |
| **AUC** |  |  |  |
| The left thyroid | 0.888 | 0.793 | 0.150 |
| The right thyroid | 0.897 | 0.746 | 0.016 |
| The isthmus of the thyroid | 0.804 | 0.625 | 0.067 |
| **Sensitivity, %** |  |  |  |
| The left thyroid | 87.5 | 73.9 | 0.350 |
| The right thyroid | 95.5 | 73.5 | 0.083 |
| The isthmus of the thyroid | 60.9 | 25.0 | 0.181 |
| **Specificity, %** |  |  |  |
| The left thyroid | 90.0 | 84.6 | 0.654 |
| The right thyroid | 84.0 | 75.6 | 0.462 |
| The isthmus of the thyroid | 100 | 100 | / |
| **Accuracy, %** |  |  |  |
| The left thyroid | 88.9 | 81.3 | 0.293 |
| The right thyroid | 87.5 | 74.7 | 0.077 |
| The isthmus of the thyroid | 87.5 | 92.0 | 0.530 |
| **PPV, %** |  |  |  |
| The left thyroid | 87.5 | 68.0 | 0.143 |
| The right thyroid | 72.4 | 71.4 | 1.000 |
| The isthmus of the thyroid | 100 | 100 | / |
| **NPV, %** |  |  |  |
| The left thyroid | 90.0 | 88.0 | 1.000 |
| The right thyroid | 97.7 | 77.5 | 0.013 |
| The isthmus of the thyroid | 84.5 | 91.8 | 0.305 |

AUC, area under the receiver operating characteristic curve; NPV, negative predictive value; PPV, positive predictive value.
