# Supplementary material for: The impact of a simple positioning aid device on the diagnostic performance of thyroid cancer in CT scans: a randomized controlled trial
Source: Cancer Imaging. 2025 May 8;25:60. doi: 10.1186/s40644-025-00878-w (PMC12063306; doi:10.1186/s40644-025-00878-w)
Supplement: Supplementary file 2 — Supplementary Material 2 [file 40644_2025_878_MOESM2_ESM.doc]

**Supplemental Table 2. The diagnostic performances in malignancy of thyroid cancer**

|  | Group A (n=72) | Group B (n=75) | P value |
| --- | --- | --- | --- |
| **AUC** |  |  |  |
| The left thyroid | 0.912 | 0.750 | 0.043 |
| The right thyroid | 0.891 | 0.706 | 0.034 |
| The isthmus of the thyroid | 0.832 | 0.673 | 0.137 |
| **Sensitivity, %** |  |  |  |
| The left thyroid | 86.4 | 60.0 | 0.149 |
| The right thyroid | 87.5 | 61.5 | 0.347 |
| The isthmus of the thyroid | 73.3 | 50.0 | 0.444 |
| **Specificity, %** |  |  |  |
| The left thyroid | 96.0 | 90.0 | 0.402 |
| The right thyroid | 90.6 | 79.6 | 0.163 |
| The isthmus of the thyroid | 93.0 | 84.6 | 0.245 |
| **Accuracy, %** |  |  |  |
| The left thyroid | 93.1 | 84.0 | 0.145 |
| The right thyroid | 90.3 | 73.3 | 0.015 |
| The isthmus of the thyroid | 88.9 | 80.0 | 0.209 |
| **PPV, %** |  |  |  |
| The left thyroid | 90.5 | 60.0 | 0.078 |
| The right thyroid | 53.8 | 61.5 | 0.908 |
| The isthmus of the thyroid | 73.3 | 33.3 | 0.067 |
| **NPV, %** |  |  |  |
| The left thyroid | 94.1 | 90.0 | 0.658 |
| The right thyroid | 98.3 | 98.3 | 0.004 |
| The isthmus of the thyroid | 93.0 | 91.7 | 1.000 |

AUC, area under the receiver operating characteristic curve; NPV, negative predictive value; PPV, positive predictive value.
